# rs2013278 in the multiple immunological-trait susceptibility locus *CD28* regulates the production of non-functional splicing isoforms

**DOI:** 10.1186/s40246-022-00419-7

**Published:** 2022-10-21

**Authors:** Yuki Hitomi, Yoshihiro Aiba, Kazuko Ueno, Nao Nishida, Yosuke Kawai, Minae Kawashima, Makoto Tsuiji, Chisato Iwabuchi, Sanami Takada, Noriko Miyake, Masao Nagasaki, Katsushi Tokunaga, Minoru Nakamura

**Affiliations:** 1 Department of Human Genetics, Research Institute, National Center for Global Health and Medicine, 1-21-1 Toyama, Shinjuku-Ku, Tokyo, 162-8655 Japan; 2 Department of Microbiology, Hoshi University School of Pharmacy and Pharmaceutical Sciences, Tokyo, Japan; 3 Clinical Research Center, National Hospital Organization (NHO) Nagasaki Medical Center, Omura, Japan; 4 Genome Medical Science Project, Research Institute, National Center for Global Health and Medicine, Tokyo, Japan; 5 The Research Center for Hepatitis and Immunology, Research Institute, National Center for Global Health and Medicine, Ichikawa, Japan; 6 Japan Science and Technology Agency (JST), Tokyo, Japan; 7 Human Biosciences Unit for the Top Global Course Center for the Promotion of Interdisciplinary Education and Research, Kyoto University, Kyoto, Japan; 8 Department of Hepatology, Nagasaki University Graduate School of Biomedical Sciences, Omura, Japan; 9 Headquarters of PBC Research in NHO Study Group for Liver Disease in Japan (NHOSLJ), Clinical Research Center, NHO Nagasaki Medical Center, Omura, Japan

**Keywords:** Immunological-trait, Genome-wide association study (GWAS), CD28, Primary functional variant, Alternative splicing, Linkage disequilibrium, CRISPR/Cas9

## Abstract

**Background:**

Ligation of CD28 with ligands such as CD80 or CD86 provides a critical second signal alongside antigen presentation by class II major histocompatibility complex expressed on antigen-presenting cells through the T cell antigen receptor for naïve T cell activation. A number of studies suggested that CD28 plays an important role in the pathogenesis of various human diseases. Recent genome-wide association studies (GWASs) identified *CD28* as a susceptibility locus for lymphocyte and eosinophil counts, multiple sclerosis, ulcerative colitis, celiac disease, rheumatoid arthritis, asthma, and primary biliary cholangitis. However, the primary functional variant and molecular mechanisms of disease susceptibility in this locus remain to be elucidated. This study aimed to identify the primary functional variant from thousands of genetic variants in the *CD28* locus and elucidate its functional effect on the CD28 molecule.

**Results:**

Among the genetic variants exhibiting stronger linkage disequilibrium (LD) with all GWAS-lead variants in the *CD28* locus, rs2013278, located in the Rbfox binding motif related to splicing regulation, was identified as a primary functional variant related to multiple immunological traits. Relative endogenous expression levels of *CD28* splicing isoforms (CD28i and CD28Δex2) compared with full-length CD28 in allele knock-in cell lines generated using CRISPR/Cas9 were directly regulated by rs2013278 (P < 0.05). Although full-length CD28 protein expressed on Jurkat T cells showed higher binding affinity for CD80/CD86, both CD28i and CD28Δex2 encoded loss-of-function isoforms.

**Conclusion:**

The present study demonstrated for the first time that *CD28* has a shared disease-related primary functional variant (i.e., rs2013278) that regulates the CD28 alternative splicing that generates loss-of-function isoforms. They reduce disease risk by inducing anergy of effector T cells that over-react to autoantigens and allergens.

**Supplementary Information:**

The online version contains supplementary material available at 10.1186/s40246-022-00419-7.

## Background

CD28 is a 44-kDa type I transmembrane protein expressed on the majority of T cells. Ligation of CD28 with ligands such as CD80 (known as B7-1) or CD86 (known as B7-2) provides a critical second signal alongside antigen presentation by class II major histocompatibility complex (MHC) expressed on antigen-presenting cells (APCs) through the T cell antigen receptor (TCR) for naïve T cell activation [1, 2]. The membrane-proximal YMNM motif and distal PYAP motif in the cytoplasmic tail of CD28 play an important role in the activation of NFAT, AP-1, and NF-κB and the subsequent transcription of interleukin (IL)-2, which influences T cell proliferation, survival, and differentiation. Without CD28 co-stimulation, IL-2 production is lost and T cells become anergic [3–5]. Therefore, CD28 acts as a positive regulator of T cell function. Cell surface expression of cytotoxic T lymphocyte–associated protein 4 (CTLA4, also known as CD152), which is highly homologous to CD28, is induced by TCR stimulation and in response to IL-2 [6]. CTLA4 binds to CD80 and CD86 with a higher affinity than CD28. This causes CTLA4 to compete with CD28 for ligand acquisition and suppresses the response of effector T cells by providing inhibitory signals that override activating signals provided by CD28 [7–9].

Mice lacking *cd28* exhibit low basal immunoglobulin levels and impaired germinal center formation, and *ctla4* was shown to produce a hyperactivated and disease-causing phenotype [10–12]. In humans, patients with loss-of-function mutations in *CTLA4* exhibit autoimmune phenotypes [13–15]. A number of studies using clinical samples have suggested that overexpression of CD80 and CD86 is correlated with the development of allergic and autoimmune diseases [16, 17]. Therefore, CD28 family members (CD28, CTLA4, CD80, and CD86) play an important role in the pathogenesis of various human diseases, especially those involving immunological traits.

The human *CD28* gene is encoded on chromosome 2q33.2. Recent genome-wide association studies (GWASs) identified *CD28* as a susceptibility gene for various immunological diseases and traits, such as lymphocyte count, eosinophil count, multiple sclerosis (MS), ulcerative colitis (UC), celiac disease, rheumatoid arthritis (RA), and asthma [18–29]. Using data from European and East Asian cohorts (10,516 cases and 20,772 controls), our research group reported the largest genome-wide meta-analysis (meta-GWAS) of primary biliary cholangitis (PBC) to date [30]. PBC is a chronic progressive cholestatic liver disease with histological features of interface hepatitis, fibrosis, ductopenia, and chronic non-suppurative destructive cholangitis. These features are due to an autoimmune reaction to the intrahepatic bile duct [31–35]. The higher concordance rate in monozygotic twins than in dizygotic twins and the higher estimated sibling relative risk suggest strong involvement of genetic factors in the development of PBC [36, 37]. PBC also showed an association with the *CD28* locus in our meta-GWAS (Table 1). Although the existence of alternative splicing isoforms of *CD28* (CD28a, CD28b, CD28c, and CD28i) was reported [38, 39], genetic variants that regulate the efficiency of alternative splicing of *CD28* have not been identified. In addition, the binding affinities of splicing isoform products to CD80 and CD86 have not been clarified.

**Table 1:** GWAS-lead SNPs in the CD28 locus for each immunological trait

Immunological trait	GWAS-lead SNP	References
Lymphocyte count	rs4675365	[18]
	rs1879877	[19]
Eosinophil count	rs4675360	[18–20]
Multiple sclerosis (MS)	rs6435203	[21]
Ulcerative colitis (UC)	rs3116494	[22]
Celiac disease	rs45620941	[23]
	rs1980422	[24, 25]
Rheumatoid arthritis (RA)	rs1980422	[24, 26–28]
Asthma	rs55730955	[29]
Primary biliary cholangitis (PBC)	rs4675370	[30]

GWAS-lead variants exhibiting the strongest associations with disease susceptibility in the *CD28* locus in GWASs are not the same among immunological traits [18–30] (Table 1). In the present study, to identify candidate primary functional variants in the *CD28* locus that contribute to various immunological traits, linkage disequilibrium (LD) mapping of GWAS-lead variants for each immunological trait was carried out using LD data for European and East Asian populations. In silico*/*in vitro functional analyses utilizing CRISPR/Cas9 gene-editing technology were then performed to identify primary functional variants. Finally, we attempted to elucidate the stability and ligand binding effect of alternative splicing isoforms of CD28.

## Results

### LD mapping with GWAS-lead variants

**Fig. 1:**
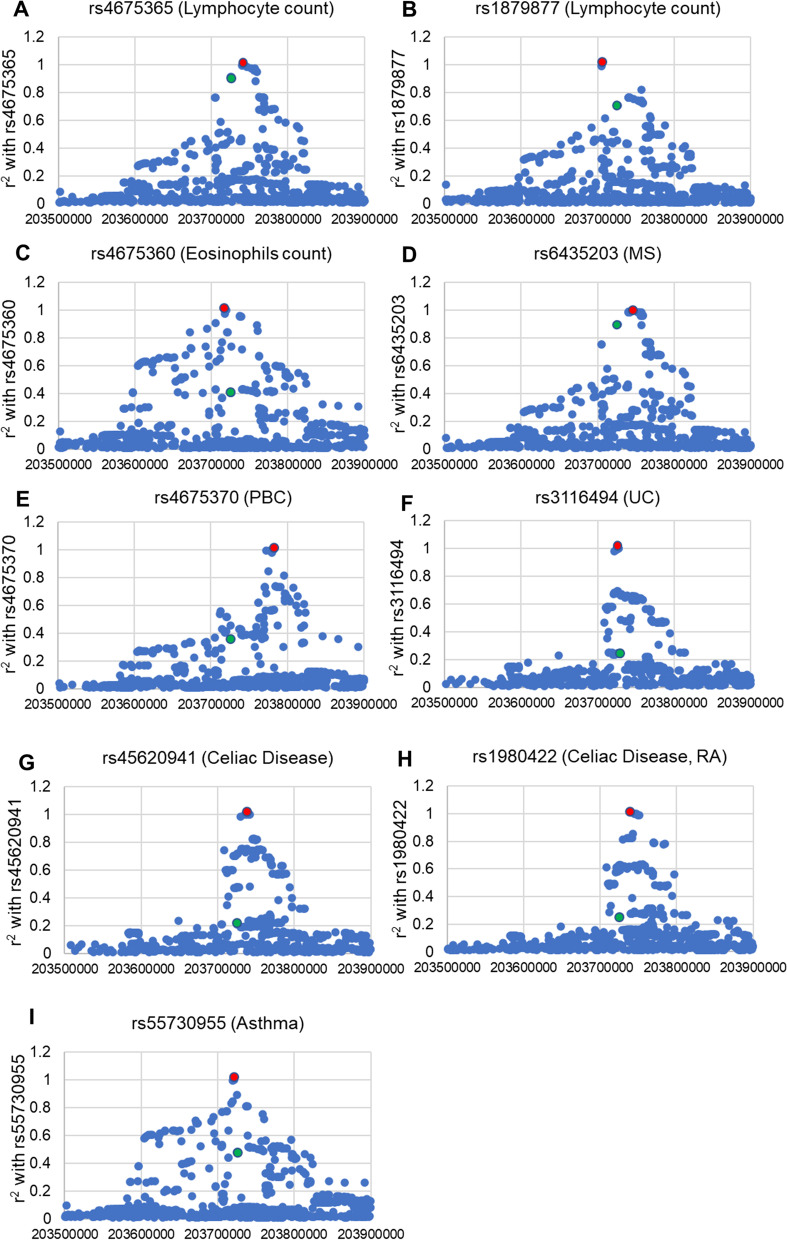
Linkage disequilibrium (LD) mapping of *CD28* SNPs with GWAS-lead SNPs (combined EAS and EUR population data). SNPs shown as red and green solid dots indicate GWAS-lead SNPs and rs2013278, respectively. Horizontal line indicates the physical position of each SNP on chromosome 2 (hg38). Vertical line indicates the r^2^ value of each SNP with GWAS-lead SNPs

A total of 157, 155, 154, 154, 135, 110, 135, 137, and 158 SNPs showed r^2^ > 0.2 with the following GWAS-lead variants, rs4675365 (associated with lymphocyte count), rs1879877 (associated with lymphocyte count), rs4675360 (associated with eosinophil count), rs6435203 (associated with MS), rs4675370 (associated with PBC), rs3116494 (associated with UC), rs45620941 (associated with celiac disease), rs1980422 (associated with celiac disease and RA), and rs55730955 (associated with asthma), respectively, by LD mapping using combined LD data for the EAS and EUR populations (Fig. 1).

**Fig. 2:**
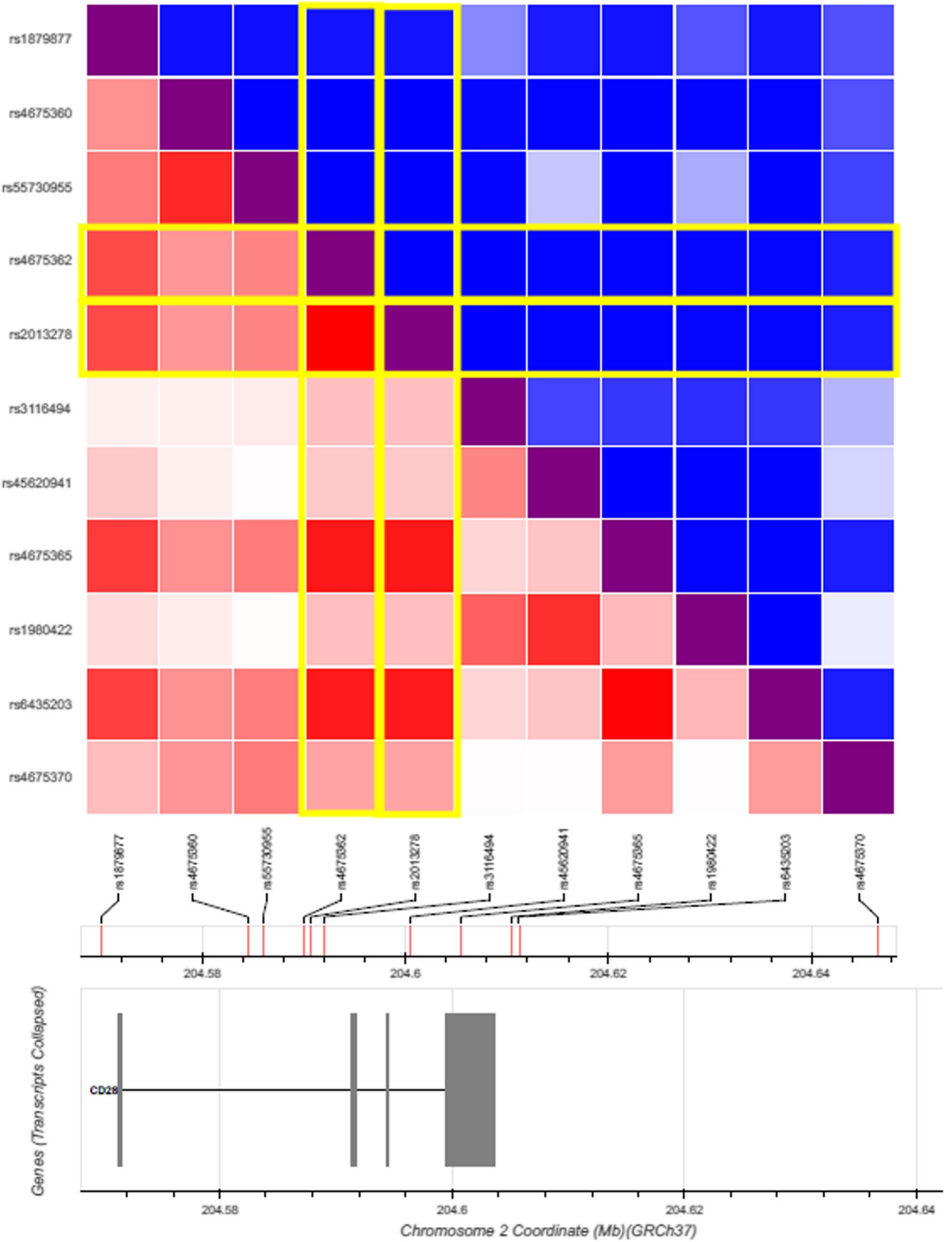
LD of rs2013278 with each GWAS-lead SNP. Densities of red and blue boxes indicate the r^2^ and D’ values of rs2013278 with GWAS-lead SNPs, respectively

Among the SNPs that showed r^2^ > 0.2 with each GWAS-lead variant, only rs4675362 and rs2013278 were shared among all immunological traits (Fig. 2, Table 2). Although the differences in LD pattern between the EAS and EUR populations were observed in six GWAS-lead SNPs (rs1879877, rs4675360, rs3116494, rs45620941, rs1980422, and rs55730955), the major ancestor in each GWAS discovery stage showed a higher r^2^ score with rs4675362 and rs2013278 than other ancestors in every GWAS-lead SNP (Additional file 1). Neither SNP was located in gene expression regulatory motifs such as H3K27Ac or the DNase high-sensitivity site (Additional file 2), nor was either associated with the expression level of *CD28* as determined by e-QTL analysis (Additional file 3). In contrast, rs2013278 was located in the third base of the Rbfox binding motif (GCATG), which is related to the regulation of splicing [40]. Similar to many genes related to the immune system [41], *CD28* reportedly encodes an alternative splicing isoform of *CD28* (CD28i) [39]. Therefore, rs2013278 was selected as a candidate primary functional variant associated with multiple immunological traits in *CD28*.

**Table 2:** Linkage disequilibrium scores (r^2^) of CD28 SNPs showed r^2^ > 0.2 with all of GWAS top-hit SNPs for 8 diseases and traits (EAS + EUR)

		lymphocyte count	Lymphocyte count	Eosinophil count	MS	PBC	UC	Celiac disease	Celiac disease, RA	Asthma
SNP	bp (Chr2: hg38)	rs4675365	rs1879877	rs4675360	rs6435203	rs4675370	rs3116494	rs45620941	rs1980422	rs55730955
rs4675362	203725285	0.9089	0.7086	0.4068	0.8993	0.3574	0.2425	0.211	0.2531	0.4799
rs2013278	203725935	0.9089	0.7086	0.4068	0.8993	0.3574	0.2425	0.211	0.2531	0.4799

### rs2013278 regulates *CD28* alternative splicing

**Fig. 3:**
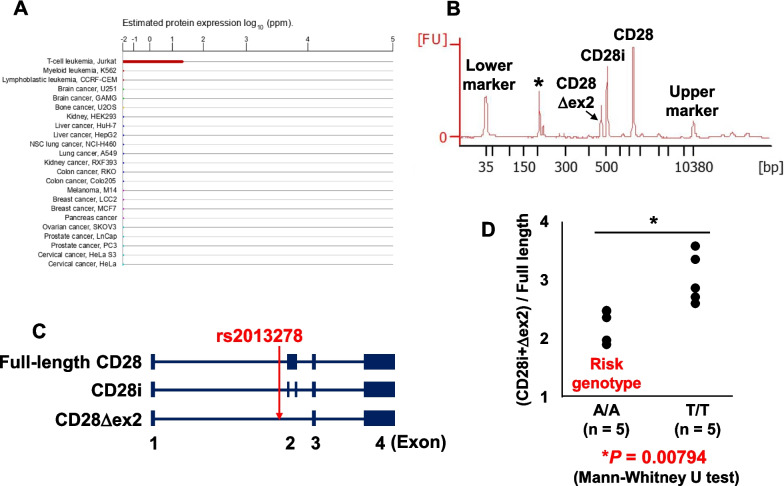
Expression of endogenous *CD28* splicing isoforms is regulated by rs2013278. **a** Endogenous expression levels of *CD28* in various human cell lines. **b** Endogenous expression levels of *CD28* splicing isoforms in Jurkat T cells. *: non-specific peak. **c** Exon–intron structure of each *CD28* splicing isoform. **d** Endogenous expression levels of CD28i and CD28Δex2 were relatively decreased in the risk allele knock-in version of Jurkat T cells generated by genome editing using CRISPR/Cas9 compared with expression levels in the non-risk allele cells

To identify the main *CD28* isoforms expressed in Jurkat T cells expressing *CD28* abundantly (Fig. 3a), RT-PCR analysis was performed. Using primers targeted within exon 1 and exon 4 of *CD28*, three amplification products were identified (Fig. 3b). By sequencing, the longer product was found to be the normal CD28 mRNA (full-length CD28; UniProtKB identifier of protein product: P10747-1), whereas the shorter products encoded alternative splicing isoforms caused by skipping of a part of exon 2 (CD28i; UniProtKB identifier of protein product: P10747-3) or a lack of all of exon 2 (CD28Δex2; UniProtKB identifier of protein product: P10747-2) (Fig. 3c). The protein products of CD28i and CD28Δex2 were thought to be deficient in a total of 85 and 119 amino acids, respectively.

Subsequently, the rs2013278 genotype knock-in versions of cell lines constructed using the CRISPR/Cas9 system were used to assess the contribution of rs2013278 to the endogenous expression levels of each *CD28* isoform. Jurkat cells were selected to knock in the rs2013278 alleles because endogenous expression of *CD28* was detected (Fig. 3a). Relative expression levels of total skipping isoforms (CD28i plus CD28Δex2) compared with full-length CD28 differed significantly between the genotype knock-in Jurkat clones of rs2013278-A/A (n = 5) and -T/T (n = 5) (P < 0.05; Mann–Whitney *U* test) (Fig. 3d). These results indicated that rs2013278 is a primary functional variant that directly regulates the alternative splicing of *CD28*.

### Expression of CD28 splicing isoforms

**Fig. 4:**
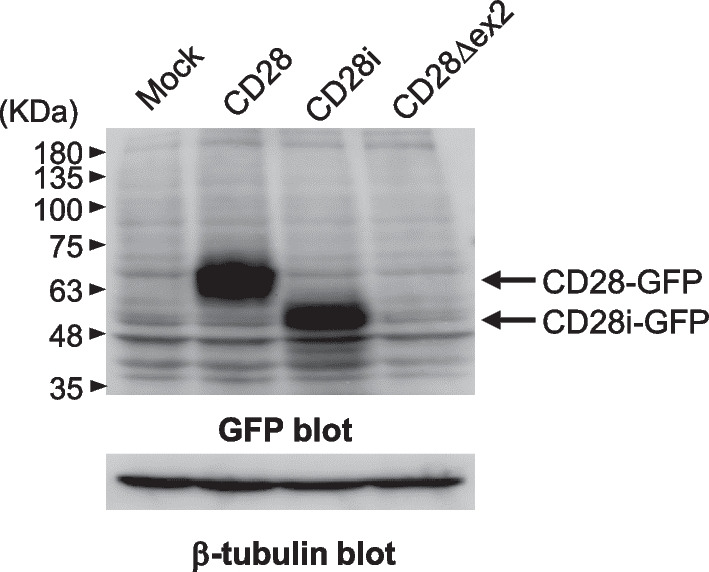
Protein expression of each CD28 splicing isoform product. Expression of GFP-conjugated CD28 isoforms was analyzed using an anti-GFP antibody. The protein product of CD28Δex2 was not detected

Because no anti-human CD28 antibody that recognizes the extracellular domain of CD28i and CD28Δex2 is currently available, protein expression of the C-terminal green fluorescent protein (GFP)-conjugated CD28 isoforms was assessed in transfectants of Jurkat cells by western blotting using an antibody against GFP. Although full-length CD28 and CD28i showed abundant protein expression in transfectant cells, CD28Δex2 did not (Fig. 4).

### Binding of CD28 splicing isoforms to the ligand CD80/CD86

**Fig. 5:**
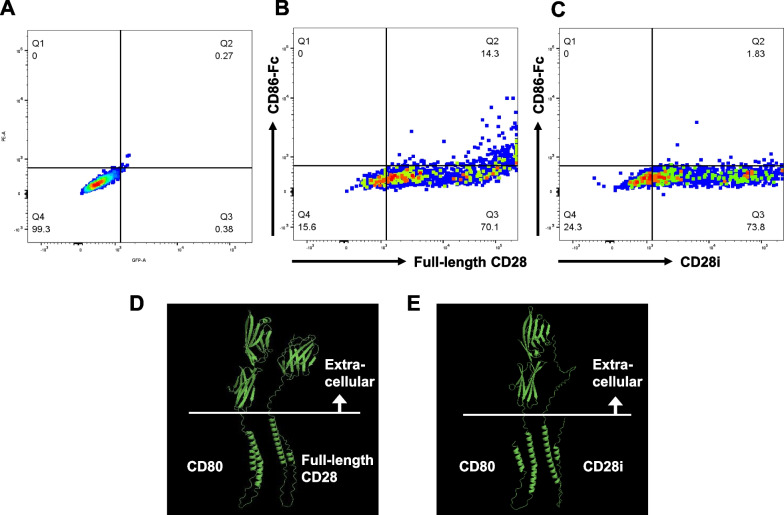
Binding of CD80 and CD86 with each CD28 splicing isoform product. **a–c** HeLa cells with transiently overexpressed GFP-conjugated CD28 and CD28i were incubated in the presence of His-tag conjugated CD86-Fc and PE-conjugated anti-His-Tag antibody. Dot plot for mock (**a**), CD28 (**b**), and CD28i (**c**). The horizontal and vertical axes indicate the expression of GFP-tagged CD28 isoforms and the bindings of His-tagged CD86-Fc, respectively. **d**, **e** In silico prediction of binding between CD80 and CD28 splicing isoforms. Although binding between CD80 and CD28 was predicted (**d**), that between CD80 and CD28i was not (**e**)

Both full-length CD28 and CD28i are reportedly located on the cell surface [39]. Although ligation of CD28 with both CD80 and CD86 provides an important second signal along with antigen presentation by the class II MHC of APCs via the TCR for naïve T cell activation [1, 2], CD86 (but not CD80) is constitutively expressed on APCs and rapidly upregulated by innate stimulation of APCs [1, 42]. Concordantly, mice lacking *Cd86* (but not those lacking *Cd80*) are unable to undergo antibody class switching and formation of the germinal center in response to adjuvant-free immunization [43]. Therefore, CD86 may play a more important role than CD80 in the initiation of immune responses. To confirm the lower binding affinity between CD28i and CD86, direct binding between C-terminal GFP-conjugated CD28i and recombinant His-tagged CD86-Fc was assessed by flow cytometry in CD28-negative HeLa cells (Fig. 3a). Cells in which full-length CD28 was strongly expressed bound directly to His-tagged CD86-Fc, but CD28i did not (Fig. 5a–c).

In contrast, as recombinant His-tagged CD80-Fc is not currently available, direct binding between CD28i and CD80 could not be examined. Therefore, the binding affinity of CD28i for CD80 was evaluated by in silico prediction. Full-length CD28 was predicted to show higher binding affinity with CD80 in their extracellular domains (DockQ score: 0.956). However, probably because most of the extracellular domain of CD28i is missing, CD28i was predicted to show lower binding affinity for CD80 (DockQ score: 0.001) (Fig. 5d, e).

Collectively, these results indicate that both CD28i and CD28Δex2 are loss-of-function splicing isoform products that reduce disease risk by inducing anergy of effector T cells that over-react to autoantigens and allergens.

## Discussion

CD28 family members, including CD28, CTLA4, CD80, and CD86, have several common structural and functional features. First, these molecules contain immunoglobulin superfamily domains in their extracellular region. The MYPPPY motif within this domain mediates the interaction between these co-stimulatory receptors and their ligands [44–46]. Second, alternative splicing isoforms have been reported in all of these genes [39, 47–49]. However, an association between the disease-related polymorphisms and alternative splicing among the CD28 family genes was reported only for *CTLA4* [47]. Therefore, the present study has demonstrated for the first time that *CD28* has a shared disease-related primary functional variant (i.e., rs2013278) that regulates the alternative splicing of CD28. The RNA sequence motif GCAUG is bound by Rbfox proteins, which are expressed in human T cells [40, 50, 51]. The Rbfox proteins reportedly inhibit hnRNP M-mediated suppression of splicing by forming a complex with hnRNP M, hnRNP H, hnRNP C, Matrin3, NF110/NFAR-2, NF45, and DDX5 [40]. rs2013278 probably alters the efficiency of alternative splicing of *CD28* by the presence (disease-risk allele) or collapse (disease-protective allele) of the GCAUG motif.

Although the primary functional variant is sometimes the same as the GWAS-lead variant (e.g., *TNFSF15* rs4979462, which is associated with PBC [52]), most other primary functional variants are not the same as the GWAS-lead variants (e.g., several SNPs associated with PBC [53–57]). In the present study, rs2013278, which was not a GWAS-lead variant, was identified as a primary functional variant in *CD28* associated with multiple immunological traits. Among GWAS-lead variants, rs4675365 (associated with lymphocyte count) and rs6435203 (associated with MS) showed stronger LD with rs2013278 (r^2^ approximately 0.9). Therefore, susceptibility to MS and changes in lymphocyte count are probably affected by the single effect of rs2013278. However, this is not the case with other immunological traits. Although rs2013278 was not associated with the *CD28* expression level, rs3116494, rs45620941, and rs1980422 showed relatively strong LD with rs13404978, which exhibited the strongest correlation with *CD28* expression level in the e-QTL analysis (rs3116494: r^2^ = 0.469; rs45620941: r^2^ = 0.575; and rs1980422: r^2^ = 0.482). Incidentally, a relatively lower r^2^ score was observed (r^2^ = 0.13) between rs2013278 and rs13404978. Another possibility is that aggregation of the effects of multiple SNPs causes the lead SNPs to show the strongest association among SNPs in the gene locus (e.g., PBC susceptibility locus *STAT4*) [58]. Therefore, immunological traits in which rs2013278 and the GWAS-lead variant show weak LD may have other primary functional variants characteristic of each disease in the *CD28* locus.

In the present study, three primarily expressed *CD28* alternative splicing isoforms (full-length CD28, CD28i, and CD28Δex2) were identified. CD28i was expressed on the cell surface [39]; however, it is incapable of binding to its ligand, CD86 (Fig. 5c). Because the total amount of the *CD28* isoforms was not associated with the genotype of rs2013278 (Additional file 3), the expression levels of the loss-of-function *CD28* isoforms (CD28i and CD28Δex2) were inversely proportional to that of full-length *CD28*. Inadequate co-stimulation of CD28 and its ligands causes hyper-reactive T cells to become anergic; therefore, relatively high expression levels of full-length CD28 associated with the disease-risk allele of rs2013278 would inhibit this anergy. This assumption is consistent with the finding that overexpression of CD86 is correlated with the development of allergic and autoimmune diseases [16, 17]. Although the other ligand, CD80, is predicted not to bind CD28i by in silico analysis (Fig. 5e), experiments examining the binding of CD28i to CD80 could not be performed because recombinant His-tagged CD80-Fc is not currently available. CD86 may play a more important role in the initiation of immune responses than CD80 [1, 42, 43]; however, the weak binding of CD28i to CD80 will need to be experimentally validated in future studies. Similarly, it will be necessary to verify the downstream signaling pathways involving CD28i, such as activation of NFAT, AP-1, and NF-κB and subsequent IL-2 transcription [3–5]. Although the Jurkat T cell line has been reported to have damaging mutations in genes involved in T cell receptor signaling (PTEN, INPP5D, CTLA4, and SYK) [59], maintenance of genome stability (TP53, BAX, and MSH2), and O-linked glycosylation (C1GALT1C1), karyotyping and genotyping of these genes were not performed in the Jurkat T cells that were used in the present study. One limitation of the present and future studies is the similarity between cell lines and normal human T lymphocytes.

The CD28Δex2 transcript was also abundantly expressed at the mRNA level (Fig. 3b); however, the protein product of CD28Δex2 was not expressed in transfectant cells (Fig. 4). Amino acid sequence changes caused by splicing sometimes significantly affect protein structure. For example, the unstable protein product of *TCF4*, which is reportedly the causal gene of an undiagnosed genetic condition, is degraded in the proteasome due to splicing-associated frameshifting [60]. In contrast, the protein product of the alternative splicing isoform of *CD72* (CD72Δex8), which is reportedly a susceptibility gene of systemic lupus erythematosus, is not degraded by the proteasome and accumulates in the endoplasmic reticulum [61, 62]. A new finding regarding protein expression of CD28Δex2 was obtained in the present study. The protein stability of CD28Δex2 is presumably lost due to the lack of amino acids encoded by exon 2.

CD28 family members are considered target molecules affecting immunological traits. To date, CTLA4 Ig (abatacept), which binds to CD80/CD86 and inhibits inflammatory T cell activation, has been approved by the US Food and Drug Administration to treat RA, juvenile idiopathic arthritis, and active psoriatic arthritis [63]. A CTLA4 super-agonist (ipilimumab) has been approved to treat melanoma [64]. Although a CD28 super-agonist (theralizumab TGN1412) caused cytokine storm in healthy volunteers in a first-in-human study [65], a clinical trial of a novel type of CD28 super-agonist (TAB08) has been performed [66]. *CD28* was identified as a disease susceptibility gene for immunological traits [18–30], and these significant associations with disease susceptibility were shown in the present study to be related to alternative splicing of *CD28*.

## Conclusion

The present study demonstrated for the first time that rs2013278, which showed stronger linkage disequilibrium with the genome-wide association study lead variants for multiple immunological traits, regulates *CD28* alternative splicing that generates loss-of-function isoforms (CD28i and CD28Δex2). They reduce disease risk by inducing anergy of effector T cells that over-react to autoantigens and allergens.

## Methods

### In silico prediction tools and databases

The UCSC genome browser [67] was used to assess the potential functional effect of candidate functional variants on transcriptional regulation (URL: http://genome.ucsc.edu/index.html).

LD data for the East Asian (EAS) and European (EUR) populations were obtained from LDlink (URL: https://ldlink.nci.nih.gov/) [68].

Data regarding *CD28* gene expression levels in various cell lines were obtained from the Human Protein Atlas (URL: https://www.proteinatlas.org/) [50, 51].

Data from the GTEx portal (version 8) were used to investigate the correlation between genotypes of all variants in the *CD28* locus and gene expression levels (URL: http://gtexportal.org/home/) [69].

Binding affinities between CD80 and splicing isoforms of CD28 were evaluated based on the DockQ score [70].

### Gene editing (CRISPR/Cas9)

Following the manufacturer’s instructions, guide-RNA (gRNA) target sequences (Additional file 4) were subcloned into pGuide-it-ZsGreen1 (Clontech Laboratories, Mountain View, CA). The transfection reagent Lipofectamine-3000 (Thermo-Fisher Scientific, Waltham, MA) was used to transfect Jurkat cells with gRNAs and donor DNAs for each allele of rs2013278 (Additional file 4). Transfected cell clones were incubated with RS-1 and SCR7 (Cayman Chemical, Ann Arbor, MI). A FACSAria II system (BD Biosciences, Franklin Lakes, NJ) was used to isolate positive clones from bulk transfectants.

After single-cell cloning, genomic DNA was extracted from cell clones using PureLink™ (Thermo-Fisher Scientific). Gene editing of target sites was confirmed using Sanger sequencing (ABI prism 3730-XL) with specific primers (Additional file 4).

### Quantitative RT-PCR

Total RNA was extracted from rs2013278 allele knock-in clones using an RNeasy kit (QIAGEN, Valencia CA). Next, we synthesized first-strand cDNAs using a High-Capacity cDNA Reverse Transcription kit (Thermo-Fisher Scientific). RT-PCR was performed using the primers shown in Additional file 5 and Ex Taq polymerase (Takara-bio, Kusatsu, Japan). Preliminary experiments showed that 33 cycles were optimal for achieving linear amplification of each *CD28* splicing isoform. Quantitation of each transcript was performed using an Agilent 2100 Bioanalyzer (Agilent Technologies, Palo Alto, CA). Differences in expression levels of *CD28* isoforms in cells of the two rs2013278 genotypes (i.e., AA and TT) were analyzed using the Mann–Whitney *U* test. These experiments were repeated 3 times with essentially identical results.

### Plasmids

cDNAs containing the entire coding region of full-length CD28, CD28i, and CD28Δex2, which do not contain nucleotides for the stop codon, were obtained by RT-PCR analysis of Jurkat cells using the specific primer pairs shown in Additional file 6. cDNAs encoding each *CD28* splicing isoform were inserted into pCR-blunt II (Thermo-Fisher Scientific) and subcloned into pAcGFP1-Hyg-N1 (Takara-bio) using *Xho*I.

### Western blotting

After transfection of the pAcGFP1-Hyg-N1 vectors, cells were lysed in RIPA buffer. Proteins in whole-cell lysates were separated by SDS-PAGE and transferred onto polyvinylidene difluoride membranes. The membranes were incubated with anti-GFP (Proteintech, Rosemont, IL) and anti-β-tubulin (Fujifilm Wako pure chemical, Osaka, Japan) antibodies. Proteins were visualized using the ECL system.

### Flow cytometry

After transfection of HeLa cells with pAcGFP1-Hyg-N1 vectors, transfectants were incubated with recombinant 6 × His-tagged human B7-2/CD86-Fc Chimera (BioLegend, San Diego, CA), followed by reaction with PE-labeled mouse anti–6xHis-tag antibody (BioLegend). Cells were then analyzed by flow cytometry using a FACSAria II and a FACSVerse system (BD Biosciences). Flow cytometry data were analyzed using FlowJo software (BD Biosciences).

## Supplementary Information

**Additional file 1:** Additional tables and figures.

## Data Availability

The datasets used and/or analyzed during the current study are available from the corresponding author on reasonable request.

## Abbreviations

APCs: Antigen-presenting cells
BCR: B cell antigen receptor
CTLA4: Cytotoxic T lymphocyte-associated protein 4
GWAS: Genome-wide association studies
GFP: Green fluorescent protein
gRNAs: Guide-RNAs
IgSF: Immunoglobulin superfamily
IL: Interleukin
LD: Linkage disequilibrium
MHC: Major histocompatibility complex
MS: Multiple sclerosis
PBC: Primary biliary cholangitis
Treg: Regulatory T
RA: Rheumatoid arthritis
TCR: T cell receptor
UC: Ulcerative colitis
