# Supplementary material for: rs2013278 in the multiple immunological-trait susceptibility locus CD28 regulates the production of non-functional splicing isoforms
Source: Hum Genomics. 2022 Oct 21;16:46. doi: 10.1186/s40246-022-00419-7 (PMC9585755; doi:10.1186/s40246-022-00419-7)
Supplement: Supplementary file 1 — Additional file 1: Additional tables and figures. [file 40246_2022_419_MOESM1_ESM.pdf]

Additional files

rs2013278 in the multiple immunological-trait susceptibility locus CD28 regulates the production of non-functional splicing isoforms

Yuki Hitomi [1,2,*], Yoshihiro Aiba [3], Kazuko Ueno [4], Nao Nishida [4,5], Yosuke Kawai [4], Minae Kawashima [6], Makoto Tsuiji [2], Chisato Iwabuchi [1], Sanami Takada [1], Noriko Miyake [1], Masao Nagasaki [7], Katsushi Tokunaga [4], Minoru Nakamura [3,8,9].

[1]Department of Human Genetics, Research Institute, National Center for Global Health and Medicine, Tokyo, Japan

[2]Department of Microbiology, Hoshi University School of Pharmacy and Pharmaceutical Sciences, Tokyo, Japan

[3]Clinical Research Center, National Hospital Organization (NHO) Nagasaki Medical Center, Omura, Japan

[4]Genome Medical Science Project, Research Institute, National Center for Global Health and Medicine, Tokyo, Japan

[5]The Research Center for Hepatitis and Immunology, Research Institute, National Center for Global Health and Medicine, Ichikawa, Japan

[6]Japan Science and Technology Agency (JST), Tokyo, Japan

[7]Human Biosciences Unit for the Top Global Course Center for the Promotion of Interdisciplinary Education and Research, Kyoto University, Kyoto, Japan

[8]Department of Hepatology, Nagasaki University Graduate School of Biomedical Sciences, Omura, Japan

[9]Headquarters of PBC Research in NHO Study Group for Liver Disease in Japan (NHOSLJ), Clinical Research Center, NHO Nagasaki Medical Center, Omura, Japan

[*]Corresponding author

Yuki Hitomi, Ph.D

Department of Human Genetics, Research Institute, National Center for Global Health and Medicine

1-21-1 Toyama, Shinjuku-ku, Tokyo 162-8655, Japan

Tel: +81-3-3202-7181 (Ext: 2869)

E-mail: yhitomi@ri.ncgm.go.jp

Additional file 1: Linkage disequilibrium scores (r^2) of CD28 SNPs showed r^2>0.2 with all of GWAS top-hit SNPs for 8 diseases and traits. (EAS and EUR)

			lymphocyte count	Lymphocyte count	Eosinophil count	MS	PBC	UC	Celiac disease	Celiac disease, RA	Asthma
ancestor	SNP	bp (Chr2: hg38)	rs4675365	rs1879877	rs4675360	rs6435203	rs4675370	rs3116494	rs45620941	rs1980422	rs55730955
EUR	rs4675362	203725285	0.928	0.854	0.637	0.92	0.392	0.763	0.56	0.735	0.119
	rs2013278	203725935	0.928	0.854	0.637	0.92	0.392	0.763	0.56	0.735	0.119
EAS	rs4675362	203725285	0.873	0.51	0.066	0.859	0.222	0.053	0.067	0.07	0.752
	rs2013278	203725935	0.873	0.51	0.066	0.859	0.222	0.053	0.067	0.07	0.752

(yellow) Major ancestor of GWAS discovery stage

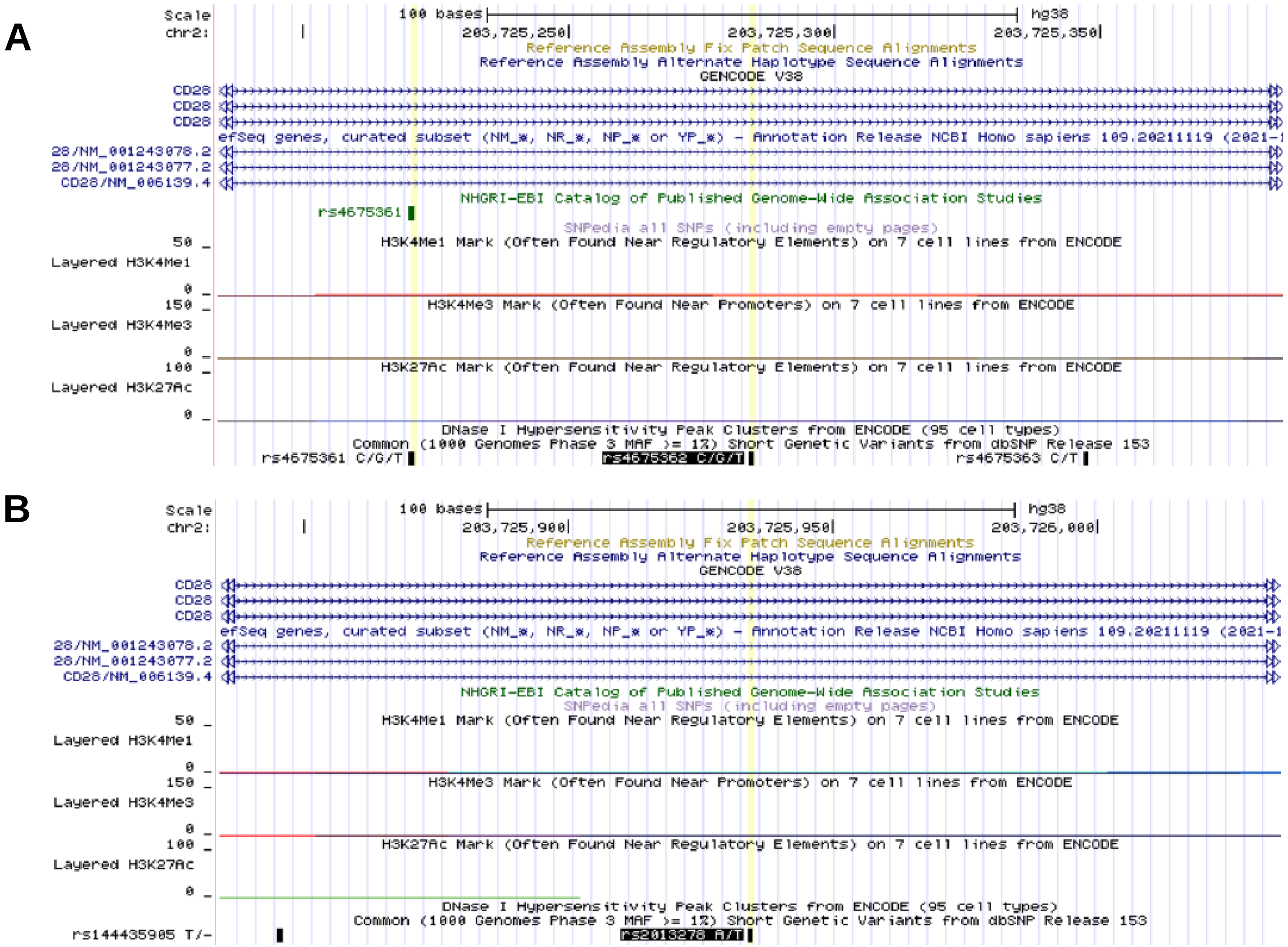

Additional file 2. Gene expression regulatory motifs around candidate primary functional variants in CD28. There were no significant gene expression regulatory motifs (H3K4Me1, H3K4Me3, H3K27Ac, and DNase high-sensitivity site) around rs4675362 (**A**) and rs2013278 (**B**).

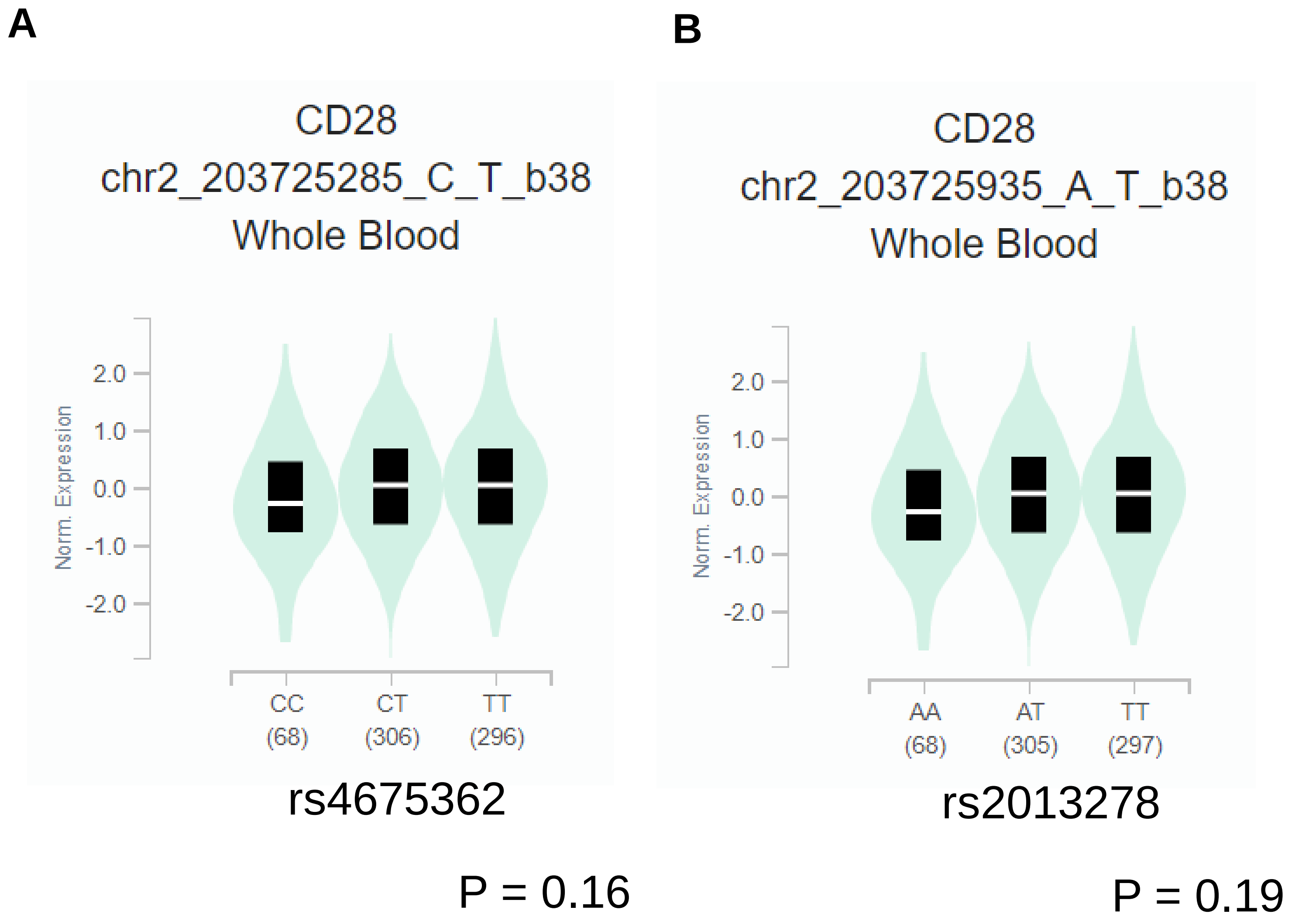

Additional file 3. e-QTL analysis of candidate primary functional variants. There was no significant association between endogenous *CD28* expression in whole blood cells and the genotypes of rs4675362 (**A**) or rs2013278 (**B**).

Additional file 4: Sequences of primers used for the synthesis of gRNAs, donor-DNA, and sequence check used for gene editing.

gRNA/donor DNA	Sequence
rs2013278-A gRNA-1 primer-F	CCGGTATTTGTAGGTTGGGCATGG
rs2013278-A gRNA-1 primer-R	AAACCCATGCCCAACCTACAAATA
rs2013278-T gRNA-1 primer-F	CCGGTATTTGTAGGTTGGGCTTGG
rs2013278-T gRNA-1 primer-R	AAACCCAAGCCCAACCTACAAATA
rs2013278-A gRNA-2 primer-F	CCGGATCTATTTGTAGGTTGGGCA
rs2013278-A gRNA-2 primer-R	AAACTGCCCAACCTACAAATAGAT
rs2013278-T gRNA-2 primer-F	CCGGATCTATTTGTAGGTTGGGCT
rs2013278-T gRNA-2 primer-R	AAACAGCCCAACCTACAAATAGAT
rs2013278 donor ssDNA-A	GATCTGACCACCTTGGCCTCCCAAAGTGCTGGGATTACAAGCATGAGGCAACATGCCCAAC CTACAAATAGATTTGTTTTTAAGTTGTGATACAGGTTT
rs2013278 donor ssDNA-T	GATCTGACCACCTTGGCCTCCCAAAGTGCTGGGATTACAAGCATGAGGCAACAAGCCCAAC CTACAAATAGATTTGTTTTTAAGTTGTGATACAGGTTT
rs2013278 sequence check-F	TCATCCCACTCTTGCAATGG
rs2013278 sequence check-R	CCACGCCCAGCTAATTTTTC

Additional file 5: Sequences of primers used for quantitative RT-PCR.

Gene name	Primer name	Sequence
CD28	CD28-F	AAGATGCTCAGGCTGCTCTT
	CD28-R	GAGATGGCGGTCATTTCCTA

Additional file 6: Sequences of primers used for subcloning.

Gene name	Primer name	Sequence
CD28	CD28_mRNA_F_XhoI	CTCGAGTAGCCCATCGTCAGGACAAA
	CD28_mRNA_R_HindIII	AAGCTTGGAGCGATAGGCTGCGAAGT
